# InterPregGen: genetic studies of pre-eclampsia in three continents

**DOI:** 10.5324/nje.v24i1-2.1815

**Published:** 2014

**Authors:** Linda Morgan, Ralph McGinnis, Valgerdur Steinthorsdottir, Gulnara Svyatova, Nodira Zakhidova, Wai Kwong Lee, Ann-Charlotte Iversen, Per Magnus, James Walker, Juan Pablo Casas, Saidazim Sultanov, Hannele Laivuori

**Affiliations:** 1 School of Life Sciences, University of Nottingham, UK; 2 Wellcome Trust Sanger Institute, UK; 3 deCODE Genetics, Iceland; 4 Scientific Centre of Obstetrics, Gynaecology and Perinatology of Ministry of Health, Kazakhstan; 5 Institute of Immunology, Uzbek Academy of Science, Uzbekistan; 6 BHF Glasgow Cardiovascular Research Centre, University of Glasgow, UK; 7 Department of Cancer Research and Molecular Medicine and Centre of Molecular Inflammation Research, Norwegian University of Science and Technology, Norway; 8 Norwegian Institute of Public Health, Norway; 9 Leeds Institute of Biomedical and Clinical Sciences, University of Leeds, UK; 10 London School of Hygiene and Tropical Medicine and University College London, UK; 11 Republic Specialized Scientific-Practical Medical Centre of Obstetrics and Gynaecology, Uzbekistan; 12 Haartman Institute, Medical Genetics, University of Helsinki, Finland

## Abstract

Pre-eclampsia is a major cause of maternal and fetal mortality in pregnancy. The identification of genetic variants which predispose to pre-eclampsia demands large DNA collections from affected mothers and babies and controls, with reliable supporting phenotypic data. The InterPregGen study has assembled a consortium of researchers from Europe, Central Asia and South America with the aim of elucidating the genetic architecture of pre-eclampsia. The MoBa collection is playing a vital role in this collaborative venture, which has the potential to provide new insights into the causes of pre-eclampsia, and provide a rational basis for novel approaches to prevention and treatment.

## Introduction

The InterPregGen consortium, a multinational collaboration between research groups from Europe, Central Asia and South America, is seeking to identify genetic factors which predispose to pre-eclampsia, a quest which has proved challenging to investigators from around the world for decades. Pre-eclampsia is the most serious of the hypertensive disorders of pregnancy, with an incidence ranging from 2-5% of pregnancies in Western Europe and North America to as high as 18% in parts of Africa [1,2]. Advances in maternity care have improved the outlook for pre-eclampsia, but it nevertheless remains a significant cause of maternal mortality even in countries with well-developed health care provision [3]. Exact figures are not available from many developing countries, but on a conservative estimate the complications of pregnancy-related hypertension account annually for over 50,000 maternal deaths worldwide [4]. Pre-eclampsia also has grave effects on the developing child, resulting in fetal growth restriction, stillbirth and sudden infant death. Expedited premature delivery contributes to perinatal morbidity, and pre-eclampsia is associated globally with an estimated 900,000 perinatal deaths annually [4].

The clinical features of pre-eclampsia are maternal hypertension and proteinuria developing after the 20^th^ week of gestation; these are manifestations of widespread vascular endothelial dysfunction. It is possible that this is a common end point to diverse pathogenetic mechanisms, but the two-stage theory is a useful model, supported by observational and experimental evidence [5]. In the first stage, inadequate invasion of maternal uterine tissues by fetal trophoblast cells results in poor vascular perfusion of the placenta. The second stage, maternal endothelial dysfunction, results from the release of damaging factors from the placenta into the maternal circulation. One such factor is sFlt-1, a soluble growth factor receptor which binds vascular endothelial growth factor (VEGF) and placental growth factor (PlGF), interfering with their function in maintaining the integrity of maternal vascular endothelium [6]. For the geneticist, an important feature of this model is the involvement of two genomes – fetal and maternal – in the pathogenesis of the disorder.

### Evidence for genetic factors

Epidemiological studies over the past five decades have provided robust and consistent support for the clinical observation that pre-eclampsia runs in families. First degree relatives of affected women have a 2- to 3-fold increase in risk of pre-eclampsia [7-10]. Furthermore, a pregnancy fathered by a man who was himself born of a pre-eclamptic pregnancy is twice as likely to be affected by pre-eclampsia [11]. A study of parous monozygotic and dizygotic female twin pairs on the Swedish Twin Register estimated the heritability of pre-eclampsia as 54% [12]. Subsequent analysis of over 700,000 births on the Swedish Birth Registry estimated pre-eclampsia heritability conferred by maternal genes as 35% (95% CI 33-36%) and that due to fetal genes as 20% (95% CI 11-24%), with similar contributions from maternally and paternally inherited genes [13]. A complex model of inheritance is likely, involving multiple genes with modest individual effect sizes.

These observations justify the search for genetic variants in mothers and babies which confer susceptibility to pre-eclampsia. Genetic studies offer an important advantage in the identification of novel pathogenetic mechanisms in diseases of pregnancy, as they circumvent the practical and ethical challenges of obtaining suitable maternal and fetal tissue early in pregnancy, when trophoblastic invasion of maternal tissues occurs; investigation of genetic variation in mother and offspring can be conducted on DNA obtained at any time. Furthermore, in a disorder such as pre-eclampsia with a rapidly progressive pathophysiology it is often difficult to distinguish between causal factors and secondary phenomena resulting from disease processes. Genetic variation which proves to be associated with disease is guaranteed to predate the disease process.

### Genetic studies of pre-eclampsia

Genome-wide linkage studies of pre-eclampsia, analysing families with two or more affected women from Australasia, Iceland, Finland and Holland, have each identified loci which appear to show linkage with pre-eclampsia in their populations, but these results have not been independently replicated [14-18]. Many candidate genes have been tested for association with pre-eclampsia, but in common with the experience in other complex disorders, these have generated inconsistent results [19]. Meta-analysis of published candidate gene studies has provided some support for genetic association with polymorphic loci in genes encoding coagulation factors II and V, the renin-angiotensin system, and factors such as methylene tetrahydrofolate reductase (MTHFR) and endothelial nitric oxide synthase (eNOS or NOS3) which play a role in maintaining endothelial integrity [20-22]. It is nevertheless noteworthy that one published meta-analysis concluded that the quality of the evidence from primary genetic association studies is frequently compromised by such factors as small study size, or inadequate genotyping quality control [20].

A major cause of the lack of success in identifying susceptibility genes has been the limited statistical power of the majority of studies performed to date. It is now apparent that many common genetic variants which predispose to complex disorders have individually small effect sizes. These variants are therefore difficult to detect by linkage analysis but can be detected with much higher power using genetic association study designs [23]. Interest is also growing in the contribution made by rare variants with larger effect sizes than those of common alleles. In either of these scenarios, the chromosomal locations of disease-predisposing variants can be detected by genetic association, but only with sufficiently large numbers of disease cases to confer adequate statistical power, and only by applying rigorously defined thresholds of statistical significance to constrain the rate of false positive results [23]. It is also of note that genetic studies in pre-eclampsia have focused on the maternal genome with relatively few studies considering the fetal genome, in spite of the epidemiological evidence for a fetal contribution to the heritability of the disorder.

The InterPregGen consortium was formed to address these deficiencies, aiming firstly to include a sufficiently large number of subjects with rigorously defined pre-eclampsia to provide adequate statistical power for association studies to detect genetic variants with small effect sizes; secondly, to use genome-wide association screening (GWAS) to scan both the maternal and fetal genomes for variants that predispose to pre-eclampsia. Also, the majority of larger published genetic studies in pre-eclampsia have been conducted in women of European ancestry, where the incidence of pre-eclampsia is amongst the lowest in the world. Therefore a further objective of the InterPregGen study was the inclusion of populations from different ethnic backgrounds in Europe, Central Asia and South America.

## The InterPregGen study design

### Subjects

InterPregGen is making use of several large biobanked DNA collections for studies of pre-eclampsia in Western Europe. These include the Norwegian Mother and Child Cohort Study (MoBa) [24,25]; the Nord-Trøndelag Health Study (HUNT) [26]; FINNPEC (The Finnish Genetics of Pre-eclampsia Consortium) [27]; GOPEC (UK Genetics of Pre-eclampsia study) [28] and deCODE Genetics’ Icelandic collection [29]. A further 4000 Central Asian mother-father-baby trios affected by pre-eclampsia are being recruited in Kazakhstan and Uzbekistan as part of the study. InterPregGen is also using genome-wide data on 2000 pre-eclamptic women and 2000 controls from the GenPE study of pre-eclampsia in Colombia, South America [30] (Table 1).

The diagnosis of pre-eclampsia is based on an international definition, requiring new-onset hypertension (systolic blood pressure ≥ 140 mm Hg; diastolic blood pressure ≥ 90 mm Hg), associated with proteinuria (≥ 300 mg/L) after the 20^th^ week of gestation [31]. Recruitment of UK, Asian subjects Central Colombian was made at the and time of diagnosis; Finnish subjects were recruited prospectively or retrospectively; MoBa subjects are selected from a prospectively-recruited pregnancy cohort; Icelandic and HUNT subjects were ascertained by linkage to medical registries and scrutiny of obstetric records.

The majority of control subjects are women with no history of hypertension (blood pressure <140/90 mm Hg) recruited during pregnancy. For Icelandic and UK subjects control data is provided by extensive existing GWAS data from population cohorts of respectively 36000 and 6000 individuals. Population control data have been used successfully for the identification of genetic associations with many complex disorders [32]; providing the condition under investigation has a low population frequency (<10%) the advantages of extensive control data more than compensate for the small reduction in statistical power resulting from misclassification of cases as controls.

All participants in the study have provided informed consent for the use of their samples, or those of their babies, in genetic studies. Approval for use of the samples and associated clinical data has been obtained from Research Ethics Committees in their country of origin, and over-arching approval for the InterPregGen study has been provided by the University of Nottingham Research Ethics Committee.

### Genome-wide association screening (GWAS)

The study is using genome-wide association screening as the primary strategy for discovery of genetic variants which are associated with pre-eclampsia. The principles of GWAS have been well-described elsewhere [33]: case and control subjects are genotyped at 600,000 to 2.5 million single nucleotide polymorphisms (SNPs) selected to capture the majority of common genetic variation across the genome. In addition to directly genotyped SNPs, thousands of additional genotypes at ungenotyped polymorphisms can be inferred by “imputation” algorithms [34] that use correlation (linkage disequilibrium) between alleles at nearby SNPs. Imputation requires knowledge of the patterns of linkage disequilibrium and for many populations these patterns are readily available from public resources such as HapMap [35] and 1000 Genomes [36]. However genome-wide linkage disequilibrium patterns are unavailable for Central Asian populations as they are not represented in publicly available databases. To supply this lack of linkage disequilibrium data from Central Asian populations, InterPregGen is undertaking whole genome sequencing of 100 Kazakh and 100 Uzbek subjects.

To maximise the statistical power of the study in a cost-effective way, InterPregGen is making use of GWAS data generated in the course of earlier studies in maternal and control samples from Iceland, the UK and Colombia. Additional GWAS is being undertaken using Illumina genome-wide arrays on offspring of pre-eclamptic pregnancies from Iceland and the UK, and also on newly collected maternal-fetal cases and controls from Central Asia. GWAS data is therefore available from a total of 7600 maternal cases and 4000 fetal cases (Table 1).

### GWAS data analysis

For each study population with GWAS genotypes, all directly genotyped and imputed SNPs are tested for disease association in pre-eclampsia mothers and babies by logistic regression assuming an additive genetic model (SNP genotypes coded 0, 1, or 2). For each study population with GWAS genotypes from pre-eclamptic mother-baby pairs, SNPs are also tested for evidence that maternal-fetal genotype interaction increases susceptibility to pre-eclampsia. Allelic odds ratios (OR) provided for each population by logistic regression analysis are combined in a meta-analysis to generate an overall OR for each SNP. The selection of SNPs for subsequent replication genotyping is based on the results of meta-analysis, with priority given to those with the lowest p-values. To adjust for the multiple statistical tests conducted in the course of GWAS, a meta-analytic p-value below 5×10^−8^ is applied for genome-wide significance (see [37] for an overview of GWAS meta-analysis methods).

### Rare variants

While the success of recent GWAS studies is impressive, a substantial part of the heritability remains unaccounted for and there are reasons to believe that variants with substantial or large effects remain undetected [38]. The selection of SNPs for commercial GWAS arrays has been largely designed to detect common allelic variants with a population frequency of >5%. There is growing interest in the contribution of rarer alleles to heritability of common disorders. InterPregGen is addressing this by taking advantage of the unique and extensive pedigree information and whole-genome sequencing data available for the Icelandic population [39], which allows for reliable detection of variants with 0.1% frequency or higher. Significant associations of pre-eclampsia with rare alleles in the Icelandic population are then tested by genotyping in other collections.

### Follow-up genotyping

A vital component of genetic studies based on genome-wide screening is replication genotyping in independent sample collections to confirm positive associations detected by genome-wide screening. The European cohorts, MoBa, HUNT and FINNPEC, are providing 2800 maternal cases, 2400 fetal cases, and 2800 controls for replication studies. The Central Asian collections will provide a further 2000 affected mothers and babies and 2000 controls (Table 1). Importantly, in the cohorts used for GWAS and for replication genotyping affected mother-baby pairs can be linked for analysis of maternal-fetal gene interactions.

Two related strategies of follow-up genotyping in the replication sets are used. A small number of SNPs with the most significant p-values on analysis of GWAS data are earmarked for fast track replication genotyping using mass spectrometry or allele-specific hybridisation technology. A larger number of SNPs with more modest evidence for association with pre-eclampsia will be evaluated by custom-designed genotyping array technology in the replication sets.

InterPregGen benefits from the availability of affected mother-father-baby trios in many of its cohorts. SNPs which are associated with pre-eclampsia susceptibility in babies are also genotyped in all available fathers of pre-eclampsia babies to determine if the ratio of allelic transmissions to PE children is different for heterozygous fathers and mothers, to test for parent-of-origin (imprinting) effects. The approach for examining parent-of-origin effects in Icelandic data will differ, due to the richness of existing Icelandic whole-genome data, providing two independent approaches to test for imprinting effects. Through whole-genome sequencing of Icelandic individuals, long-range haplotype phasing and parental-origin determination the deCODE dataset provides complete information of the parental-origin of each allele, without additional genotyping of paternal samples.

## Clinical translation

We anticipate that the research effort will identify functional DNA variants, or their proxies, which pre-dispose to pre-eclampsia and its complications, leading to enhanced understanding of the underlying disease process. InterPregGen will explore the potential for translating this information into clinical benefits through risk-profiling and the development of novel predictive markers. Currently the prediction of women at high risk of pre-eclampsia depends on clinical criteria, including the identification of risk factors at antenatal booking [40]. These include primiparity, multiple pregnancy, a previous history of pre-eclampsia, obesity, and evidence of hypertension, diabetes or renal disease prior to pregnancy. Even amongst these high-risk women only a minority will develop pre-eclampsia (about 15%), and better predictive models are required. The potential for genetic profiling in identifying individuals at risk of complex disorders has not yet been realised for the majority of diseases; this may reflect the incomplete catalogue of genes associated with these disorders. In the case of pre-eclampsia, it is possible that both maternal and paternal genotypes are of importance. InterPregGen will identify a panel of maternal and paternal genotypes and examine their value as “stand-alone” risk markers, and in predictive profiles in combination with clinical risk factors. In this regard the availability of extensive and reliable clinical information collected during pregnancy will be of vital importance in the evaluation of predictive models. The identification of metabolic pathways implicated by susceptibility genes in the pathogenesis of pre-eclampsia will highlight metabolites which are likely targets for the development of plasma or urinary biomarkers as cost-effective alternatives to genetic profiling.

## Role of MoBa in the InterPregGen study

MoBa is a unique collection of samples and data gathered prospectively at the time of pregnancy. For researchers into the genetics of complex disorders of pregnancy such as pre-eclampsia MoBa offers the advantages of a searchable database of reliable clinical information which can be used to identify cases and controls which fulfil the inclusion criteria of the study with a high degree of confidence. The well-managed biobanking facility ensures that DNA of dependable quality is available for genotyping studies from large numbers of cases and controls, a vital requirement for adequately powered genetic association studies. In contrast to many case-control collections, MoBa includes data and DNA samples from the partners and babies of pregnant women, which will enable InterPregGen to explore both maternal and fetal genomes, the interactions between maternal and fetal gene variants, and the contribution of paternal genes to pre-eclampsia. The biobanking expertise of MoBa partners in the InterPregGen study is proving invaluable in the discussion of ethical policies, and in the development of procedures for the newly formed biobanks in Uzbekistan and Kazakhstan.

## Conclusion

The identification of genetic factors which predispose to pre-eclampsia is a large undertaking, in which MoBa has a key role. InterPregGen is conducting the largest ever genome-wide association screen of maternal and fetal genes and their interactions in this disorder, and is also the first with adequate power to investigate the role of genetic imprinting (parent-of-origin effect). The ability to compare the genetic architecture of pre-eclampsia in subjects from three continents may give insights into the source of variation in the frequency of the condition across different ethnic groups. The prospects are good for novel findings which will ultimately translate into a better understanding and management of a condition which remains one of the major killers of pregnant women and their babies throughout the world.

## Figures and Tables

**Table 1:** Sources and number of samples available for genotyping in the InterPregGen study. GWAS: genome-wide association screening.

Biobank	Country of origin	Maternal PE cases	Fetal PE Cases	Controls
Samples for GWAS				
GOPEC	UK	1800	1000	6000
deCODE	Iceland	1800	1000	36000
Kazakhstan	Kazakhstan	1000	1000	1000
Uzbekistan	Uzbekistan	1000	1000	1000
GenPE	Colombia	2000	–	2000
**Total samples for GWAS**		**7600**	**4000**	**46000**
Samples for follow-up genotyping				
MoBa	Norway	1200	1200	1200
HUNT	Norway	1000	600	1000
FINNPEC	Finland	600	600	600
Kazakhstan	Kazakhstan	1000	1000	1000
Uzbekistan	Uzbekistan	1000	1000	1000
**Total samples for follow-up**		**4800**	**4400**	**4800**
